# Quasi‐1D Conductive Network Composites for Ultra‐Sensitive Strain Sensing

**DOI:** 10.1002/advs.202403635

**Published:** 2024-06-28

**Authors:** Zhiyi Gao, Dan Xu, Shengbin Li, Dongdong Zhang, Ziyin Xiang, Haifeng Zhang, Yuanzhao Wu, Yiwei Liu, Jie Shang, Run‐Wei Li

**Affiliations:** ^1^ CAS Key Laboratory of Magnetic Materials and Devices Ningbo Institute of Materials Technology and Engineering Chinese Academy of Sciences Ningbo 315201 P. R. China; ^2^ Zhejiang Province Key Laboratory of Magnetic Materials and Application Technology Ningbo Institute of Materials Technology and Engineering Chinese Academy of Sciences Ningbo 315201 P. R. China; ^3^ College of Materials Science and Opto‐Electronic Technology University of Chinese Academy of Sciences Beijing 100049 P. R. China; ^4^ Institute of Micro/Nano Materials and Devices Ningbo University of Technology Ningbo City 315211 P. R. China; ^5^ School of Future Technology University of Chinese Academy of Sciences Beijing 100049 P. R. China

**Keywords:** acoustic perception, conductive composites, flexible strain sensor, percolation effect, ultra‐sensitive

## Abstract

Highly performance flexible strain sensor is a crucial component for wearable devices, human‐machine interfaces, and e‐skins. However, the sensitivity of the strain sensor is highly limited by the strain range for large destruction of the conductive network. Here the quasi‐1D conductive network (QCN) is proposed for the design of an ultra‐sensitive strain sensor. The orientation of the conductive particles can effectively reduce the number of redundant percolative pathways in the conductive composites. The maximum sensitivity will reach the upper limit when the whole composite remains only “one” percolation pathway. Besides, the QCN structure can also confine the tunnel electron spread through the rigid inclusions which significantly enlarges the strain‐resistance effect along the tensile direction. The strain sensor exhibits state‐of‐art performance including large gauge factor (862227), fast response time (24 ms), good durability (cycled 1000 times), and multi‐mechanical sensing ability (compression, bending, shearing, air flow vibration, etc.). Finally, the QCN sensor can be exploited to realize the human‐machine interface (HMI) application of acoustic signal recognition (instrument calibration) and spectrum restoration (voice parsing).

## Graphical abstract

A “quasi‐1D” conductive network structure and a highly sensitive force‐sensitive sensing unit are proposed for application in ultra‐high sensitivity strain sensors, with high sensitivity (GF > 106) at 0.01% strain.

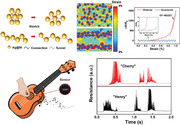

## 1 Introduction

With the flourishing development of artificial intelligence (AI) and Internet of Things (IoT), there is a growing demand for designing and utilizing flexible mechanical sensors with high sensitivity, reliability, and durability.^[^ ^1^, ^2^, ^3^, ^4^, ^5^, ^6^, ^7^, ^8^, ^9^, ^10^ ^]^ Especially the flexible strain sensors can respond to subtle mechanical deformations and provide electrical output. The flexible ultra‐sensitive strain sensor can be easily integrated into wearable devices for the achievement of human motion detection, epidermal health monitoring, and soft robotics, which will likely be applied as the key technologies of virtual reality and augmented reality (VR/AR).^[^ ^11^, ^12^ ^]^ The ultrahigh sensitivity is one of the most conclusive performance metrics and finally determines the practical applications, such as the HMI may require the fast and real‐time high‐precision of acoustic signals. The facile fabrication of highly performance strain sensors is of great importance for the construction and development of smart sensing systems.

Usually, the combination of conductive materials and flexible substrates is the intuitive method for designing flexible strain sensors.^[^ ^13^ ^]^ Recently, functional sensing materials including low dimensional materials such as nanoparticles,^[^ ^14^ ^]^ metallic nanowires,^[^ ^15^ ^]^ carbon nanotubes,^[^ ^16^ ^]^ and 2D materials like MXene^[^ ^17^ ^]^ have been widely exploited to fabricate highly sensitive strain sensors due to their excellent electrical and mechanical properties. The sensitivity of flexible strain sensors mainly on the contact separation of sensitive materials under strain or the resistance change caused by the destruction of conductive network structure. Most researchers have focused on the special design of the conductive network structure of the active layer. For example, researchers designed programmed crack structure,^[^ ^18^ ^]^ in situ micro‐convex structure,^[^ ^19^ ^]^ and segregated filler conductive network structure^[^ ^20^ ^]^ to improve device sensitivity. However, these specific structural designs often require specific templates or sophisticated manufacturing processes.^[^ ^21^ ^]^ Besides, the dimension incompressibility of the strain‐sensitive structure, detachment problems are likely to occur among the layer interface, eventually leading to structural and electrical failure, which results in the deprivation of large‐scale application. On the contrary, conductive percolation composites (CPCs) usually have only one single sensing layer to enhance the performance of sensors. The strategy of constructing a conductive percolation network is facile and reproducible, and the percolation threshold of the CPCs is greatly influenced by the distribution and geometry of the fillers.^[^ ^22^ ^]^ Yang et.al.^[^ ^23^ ^]^ demonstrated that the strain sensor with filler fraction just above the percolation threshold of Ag NFs/latex composites exhibits high sensitivity to the applied strain. Nevertheless, limited by the morphology of the conductive network and its interfacial bonding with the matrix materials, the whole composites still maintained electrical continuity under deformation. In other words, the multiple permeable paths of the conductive network make the conductive composite material still have multiple conductive paths under the condition of small strain, so that the sensitivity of the sensor depends on the large strain of the substrate.^[^ ^24^ ^]^ Accordingly, reducing the permeability path and transforming the conductive network into a quasi‐1D structure can rapidly reduce or even eliminate the conductive path under small strain, effectively improving the sensitivity. However, the strain sensor based on a percolation conductive network with ultra‐high sensitivity under strain <1% has not been reported.

In this work, we reported an ultrasensitive sensor based on a quasi‐1D conductive network (QCN). Based on the properties that silver nickel particles (Ag@Ni) can be adjusted and oriented under a magnetic field, the directional conductive quasi‐1D network composite was constructed by combining the thermoplastic polyurethane (TPU). The dimensional of the whole connected percolation network is reduced to along the direction magnetic line of force. Due to the heterogeneous modulus structure of the QCN‐based composites, stress tends to concentrate along the ordered metal particles instead of the TPU matrix region with lower modulus. The ordered conductive network effectively confines the tunneling modulated charge transfer through the rigid metal particles. The QCN composites have an ultra‐large gauge factor (GF) of up to 862 227 under the strain of 1%, which is two orders of magnitude higher than the unordered conductive composites. Besides, the ultra‐sensitive strain sensor based on QCN composites shows an ultra‐low detection limit (0.01%) and fast response time (24 ms). To demonstrate the strategy of improving sensitivity and explain the mechanism of the QCN composites, a finite element model was proposed to simulate the special percolation network structure evolution under strain. The strain transfer process and the particle distance change factor (DF) of the composites effectively validated the exponential decrease of the tunneling current under the strain sensing process. Furthermore, the QCN composites can be exploited as multi‐type subtle stimuli (compression, bending, airflow, etc.). The outstanding characteristic of the ultra‐sensitive sensor can be utilized as a voice recognition application for HMI.

## 2 Results and Discussion

### 2.1 The Design Principle and Mechanism of the QCN Composites

**Figure 1:**
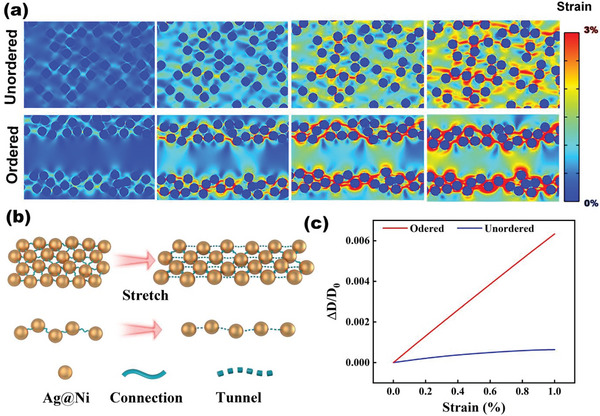
The principle of quasi‐1D conductive network (QCN) and the sensing mechanism. a) The difference of dynamic strain distribution based on unordered and ordered conductive networks at the stretching strain of 1%. b) Schematic illustration of the structural evolution of the QCN and close‐packing conductive network under strain. c) The average distance variation of the Ag@Ni particles under the strain of 1%.

The highly sensitive conductive network structure should often be properly regular or even separated which enables large electrical response under small deformation.^[^ ^20^, ^25^ ^]^ The strain propagation process of the rigid conductive particles in elastic substrates was visualized in **Figure** **1**a. Under the uniaxial tensile loading, the orderly arranged hard inclusions (Ag@Ni) in the soft matrix (TPU) notably regulated the local stain field and its propagation direction while the unordered composites showed heterogeneous deformation. Additionally, strain is preferentially released between particles along the tensile direction instead of randomly dissipating in the substrate. This phenomenon indicates a valid strategy to enhance sensitivity via the orientation of conductive filler. As illustrated in Figure 1b, the sensing mechanism of percolative network composites is derived from the strain‐dependent morphology and structure change. After stretching, the neighboring particles may lose contact and lead to the breakage of percolation pathways which represents the large resistance‐strain effect.^[^ ^26^ ^]^ However, the conductive particles are inclined to aggregate spontaneously and strongly disordered due to the minimization of surface energy, which endows the initial electrical connection of the whole composites.^[^ ^27^ ^]^ The particle arrangement can be described as We considered that the tightest packing arrangement of circles in a 2D.

Besides, due to Poisson's effect on stretchable material, the adjacent particles perpendicular to the direction of strain could compress together for conductance retention. So, the number of parallel percolation pathways is the predominant factor of sensitivity. In other words, the GF, the maximum sensitivity will reach the upper limit when the whole composites remain only one percolation pathway.^[^ ^28^ ^]^ The quasi‐1D conductive network can reduce redundant percolation pathways, and effectively define the tunneling spread along uniaxial tension through the Ag@Ni network. We calculated the average distance change among adjacent nanoparticles within the network for objective quantitative analysis of the correlation between strain and resistance. First, we considered that the tightest packing arrangement of circles in a 2D plane is the hexagonal close‐packed arrangement. The average distance (*D*) between the center circle and its nearest six circles was extracted from the FEA model. The detailed derivation is described in Note S1 (Supporting Information). As the results of Figure 1c, the relative distance change of the ordered network is significantly higher than that of the unordered network under the same deformation of 1%. The distance increase will ultimately yield an exponential growth of tunneling current under the voltage applied.^[^ ^29^ ^]^ The huge strain resistance effect of the QCN composites is expected to solve the strain‐sensitivity dilemma of percolative network composites.

### 2.2 The Fabrication and Morphology of the QCN Composites

**Figure 2:**
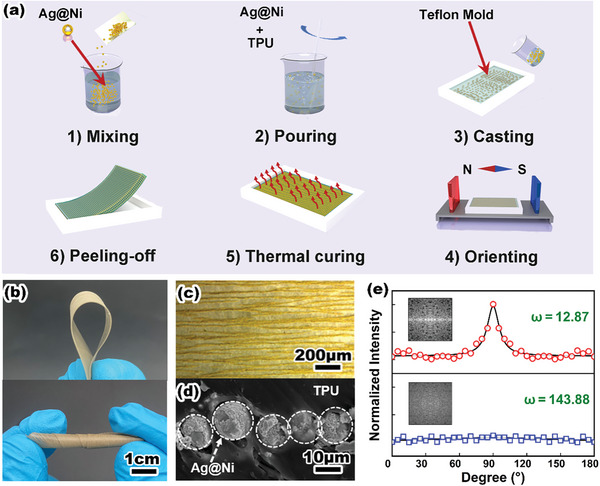
The preparation and characterization of the QCN composites. a) Schematic illustration of the preparation of QCN composites. b) Optical image of the flexible QCN composite film. c) The quasi‐one chain from the top side optical image view of the QCN composites film. d) The single quasi‐one chain consists of several Ag@Ni particles from the section SEM image. e) The Corresponding 2D FFT image and alignment plots from the ordered and unordered conductive composites film.

According to the designing strategy, the QCN composites could be optimized by field induction ordered conductive composites. Here we realized the controlled arrangement of silver–nickel particles under the guide of a magnetic field, as shown in **Figure** **2**a. The schematic diagram of the components of the magnetization equipment is illustrated in Figure S1 (Supporting Information). The QCN composite is mainly composed of an elastomer matrix and an orderly arrangement of conductive particles embedded in it. The thermoplastic polyurethane (TPU) solution was pre‐prepared to fully disperse the Ag@Ni particles, which were synthesized by silver mirror reaction with an average diameter of 15 µm (Figure S2, Supporting Information). The optical microscope images of Figure S3 (Supporting Information) indicated that the random composites without any external assistance tended to reunite together quickly due to the interactions within the magnetic particle. The assembly of conductive particles can be accomplished rapidly as the guidance of a magnetic line provided by the parallel block magnet (≈150–200T distributed in the middle of the Teflon mold). After the thermal curing molding process, the Ag@Ni particles ultimately maintain the orderly distribution status inside the matrix. The thickness of QCN composite material is ≈100 µm, exhibiting mechanical flexibility that can be easily folded and twisted. (Figure 2b). Through the stress‐strain test Figure S4 (Supporting Information), the elongation at break of the QCN composites was up to 813%, and Young's modulus was calculated as 3.03 MPa. From the top surface view of the ordered conductive composites optical image (Figure 2c), the orderly distributed magnetic conductive particles along the lines of the magnetic force served as pathways for electron conduction, every pathway retained a certain distance while also binding with each other for the integral feedthrough of the electron, which is the same microstructure as typical morphology under a magnetic field.^[^ ^30^ ^]^ A cross‐section SEM image (Figure 2d) of an ordered conductive composite (Figure 2c) shows that Ag@Ni arranges particles radially in elastomers. This was also verified by the simultaneous presence of Ag and Ni elements in the EDS mapping (Figure S5a–d, Supporting Information). The spacing of the linear structure was mainly determined by the distribution of magnetic lines of force. The greater the intensity of the magnetic field which means the denser the distribution of magnetic lines of force, resulting in the decrease of the liner spacing. Furthermore, to evaluate the orientation degree of the QCN chain along the magnetic lines, the grayscale pixel intensities of conductive composite optical microscopic images (ordered and unordered) are plotted as 2D (fast Fourier transform) FFT alignment plots (Figure 2e) against the corresponding angles among 0–180°. Statistically based on the unordered conductive composite, there is almost no change according to the vertical axis value at all angles. Conversely, the frequency reached the peak value at 90°, which revealed the conductive pathway orderly arranged along the axial direction. Furthermore, these two types of pictures statistically showed a considerable difference in distribution (ω = 12.87, 143.88). The composite with a directional conductive pathway formed along the orientation direction will show greater electrical structural changes when subjected to stress and finally exhibit ultra‐sensitive sensing performance. The characterization based on the alignment of the material provides a guiding significance for the electrical mechanism of the deformation mechanism during stretching. This characterization method based on optical microscopy images can further select the QCN composites with ultra‐sensitivity.

### 2.3 Sensing Performance of the QCN Composites

**Figure 3:**
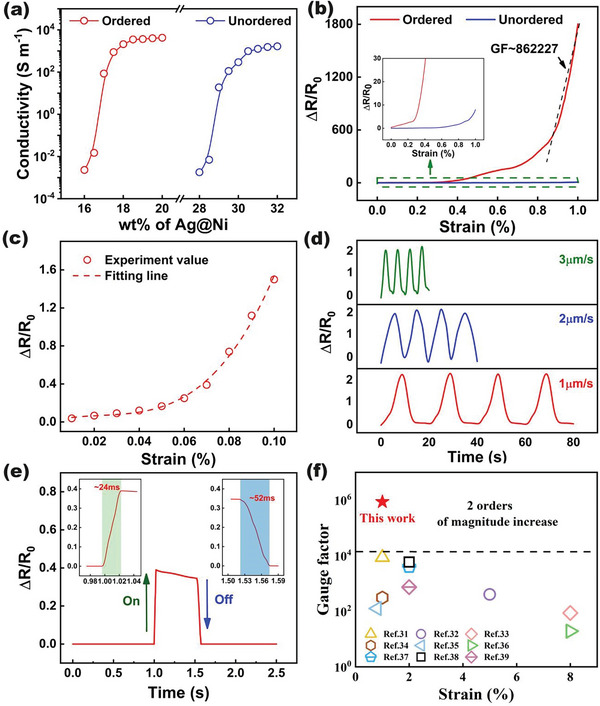
The basic electrical property and sensing performance of QCN composites. a) Electrical conductivity of the ordered and unordered conductive composites. b) Relative resistance changes of the ordered and unordered conductive composites under the strain of 1%. c) The resistance response of the QCN composites within the 0–0.1% strain range. d) The relative resistance changes of the QCN composites under the strain of 0.1% of different stretching rates. e) The response time of the QCN‐based sensor. f) Comparison of the sensitivity (GF) with previously reported sensor within the strain of 10%.

Although the percolative pathway number of the QCN composites is greatly reduced, the conductivity of the QCN composites is still maintained at 10^4^ S cm^−1^ level. We can calculate the corresponding conductivity of the film at different loading values according to the initial resistance (*R*_0_). We can calculate the relative conductivity of the film at different composite contents, described as:

(1) $$\sigma_{0}=\frac{L}{R_{0}S}$$

where *L* and *S* is the length and cross‐sectional area of conductive film. As shown in **Figure** **3**a, the electrical conductivities of the composites for both ordered samples and unordered samples rise with the increasing volume fraction of the conductive filler. When the mass fraction of the filler reached the percolation threshold, the whole composite could achieve an insulated‐conductive transition. The percolation threshold of the ordered composites is much lower than the percolation threshold of the composites. This verified that the percolation threshold can greatly decrease by the directional arrangement of the conductive fillers. This is mainly because the conductive particles only need to be distributed in the spaced polymer substrate to form a conductive pathway, and will not be irregularly distributed in the matrix, thus greatly reducing the percolation threshold of the composites. Previous studies have shown that filler fraction just above the percolation threshold of conductive composites exhibits high sensitivity under deformation.^[^ ^21^ ^]^ Thus, we selected the relevant composite films (18 wt.%, 30 wt.%) for the subsequent sensor performance test. The crucial parameter of the sensor was defined as Gauge Factor (GF)$=(\frac{R-R_{0}}{R_{0}})/$ε, where R, R_0_, and ε denote the resistance under tensile strain, initial resistance, and applied strain. The relative resistance change of the QCN composite was obviously higher than the original unordered composites at the strain of 1%, the liner curve fitting results in Figure 3b indicated that the sensitivity of the QCN sensor is up to 862 227. The resistance variations of the composite were mastered by the distance of the adjacent particles. Here, we proposed a simple model to describe the exponential growth strain‐resistance curve. The internal structure of the piezoresistive material can be equivalent to the collection of numerous resistor units (m × n), as Figure S6a (Supporting Information) shows the schematic structure. During the loading process, the connected resistor will gradually break into the isolated domain, eliminating the pathway for electrons and increasing the resistance. The ordered conductive composite sensor can be regarded as a series circuit that could be disconnected easily. The sensor based on an unordered conductive composite is considered a density conductive interconnection network as a series & parallel circuit (Figure S7b, Supporting Information). The total particle number (*m*) and the total percolative pathway (*n*) can describe the whole conductive structure. To be more intuitively, the initial resistance of the both circuit related parameters can be defined equally as:

(2) $$initial\frac{1}{R}=n\frac{1}{mR_{0}}\to R=\frac{mR_{0}}{n}$$

We define *f* as a function to indicate the remaining connection lines; the resistance after stretching is as follows:

(3) $$R_{\mathrm{unordered}}=\frac{mR_{0}}{nf(\varepsilon )}$$

(4) $$R_{\mathrm{ordered}}=\frac{mR_{0}}{nf{\left(\varepsilon \right)}^{m}}$$

The series and parallel circuits can perfectly fit the *R*–ε curve trend difference of the ordered and unordered composites. Since the total particle number (*m*) is considerably large positive integers, the resistance‐strain curve on statistics shows considerable disparity between the ordered and unordered composites.

The resistance response remained identical after every stepped slight strain change (0.01%), reflecting the sensing capability to distinguish the 0.1% strain difference of the sensor (Figure 3c). As shown in Figure 3d, the relative resistance value of QCN remains unchanged at different stretching rates (1, 2, and 3 µm), verifying the sensing stability and feasibility of the sensor. The response and recovering time were estimated as 24 and 52 ms, respectively (Figure 3e), which is faster than tens of milliseconds reported in most literature. The fatigue performance of the QCN sensor was also evaluated, as shown in Figure S6 (Supporting Information). The relative resistance changes showed volatility at first and eventually stabilized at the same level, which may arise from the reset process of the fillers and the relaxation of the polymer matrix. We compared the GF of the QCN sensor with other highly sensitive strain sensors, the sensitivity showed at least two orders of enhancement (Figure 3f).^[^ ^23^, ^24^, ^31^, ^32^, ^33^, ^34^, ^35^, ^36^, ^37^ ^]^ The state‐of‐art performance of the QCN composites perfectly verified the strategy of designing ultra‐sensitivity strain sensor by orientation of conductive filler (Table S1, Supporting Information).

**Figure 4:**
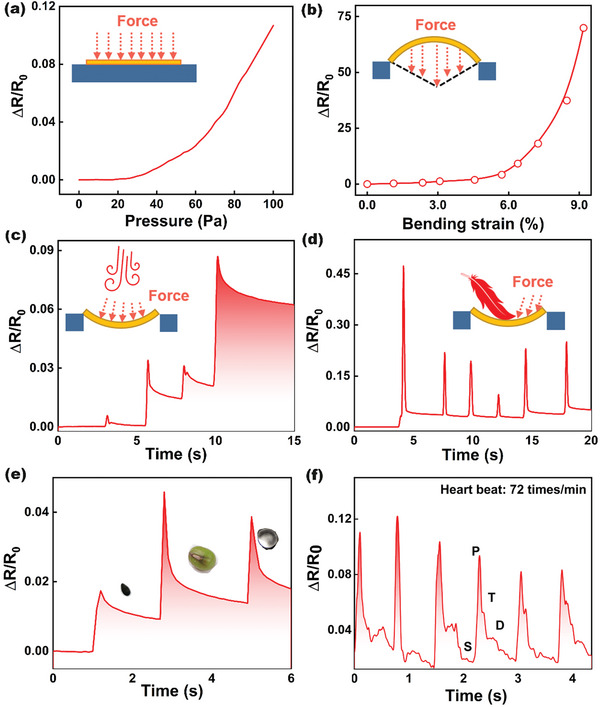
The multi‐mechanical stimuli perception of the QCN sensor. a,b) Relative resistance changes of the QCN sensor under pressure and bending loading. c) The resistance response of the QCN sensor to subtle pressure induced by airflow. d) The resistance response of the QCN sensor to the shearing force caused by feather swiping. e) Relative electrical response of the QCN sensor to several light object loading. f) Relative electrical response of the QCN sensor to human pulse.

The QCN composites exhibited superior sensitivity to various mechanical stimulus perceptions. First, The QCN sensors are capable of detecting pressure, with sensitivities of S = 5 kPa^−1^ at 0–100pa (**Figure** **4**a). The bending test was systematically investigated as the inset diagram of Figure 4b, and the bending can be converted as ε  =  6*Sh*/*L* ^2^,where s is the deflection, h is the sample thickness, and L is the span. The resistance of the QCN sensor increased monotonously as the bending strain increased. When the bending strain was up to 9%, the calculated bending angle was ≈40°, which can fulfill the requirement of most bending application scenarios. Figure 4c shows that the QCN sensor can detect the weak vibration induced by airflow, the different flow angles toward the sensor surface could also be distinctions. Besides, the QCN sensor also had an obvious recognition ability for the shear force generated by feathers scratching the surface (Figure 4d). The slight weight difference of the sesame, green bean, and TPU particles can also be easily distinguished by the QCN sensor (Figure 4e). The excellent mechanical perception ability of the QCN sensor can be exploited as a flexible pulse sensor for fidelity detection of the small deformation of the epidermis (Figure 4f).

### 2.4 Application of Ultra‐Sensitive Flexible Strain Sensors

**Figure 5:**
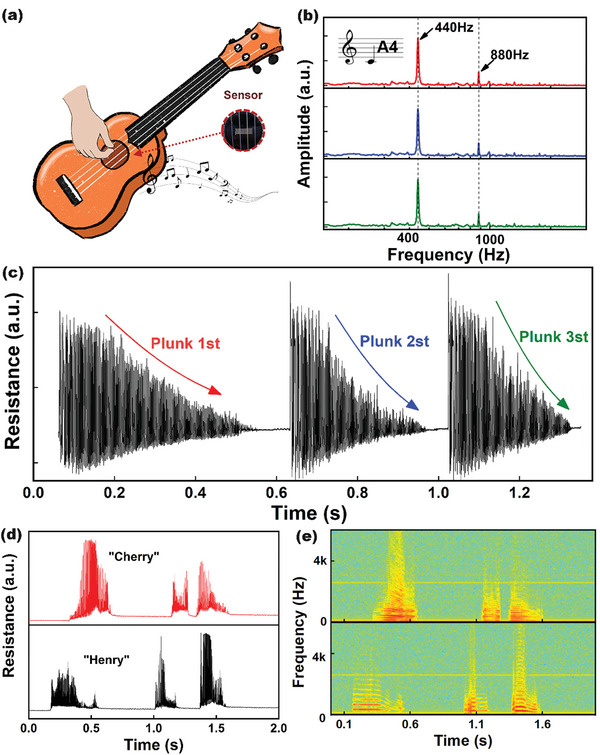
The sound detection and voice recognition application of the QCN sensor. a) Schematic diagram of the ordered conductive composite sensor as a Ukulele tuner. b) The FFT analysis signal of the sensor for the detection of note A4 (repeated 3 times) c) The real‐time resistance changes during continuous three‐wave string fluctuations. d,e) The time domain and frequency domain of the voice signal recorded by the QCN sensor.

The ordered conductive structure enables the QCN sensor noticeable sensitivity and fast response ability. This may provide possibilities for the accurate identification of weak mechanical vibrations like acoustic waves. Acoustic waves are characterized by attenuation with the propagation of a medium and by decoding information such as frequency and intensity from acoustic signals. To demonstrate such a capability, we modulated a series of sinusoidal waveforms by software so as to exert mechanical vibration over a wide frequency range and density distribution. As the diagram is shown in Figure S9a (Supporting Information), a loudspeaker was employed to emit sound waves over the different distances of air propagation. The PET frame with a hole inside was designed to fix and provide vibration space for the sensor. Propagation of airflow vibration (Figure S9b, Supporting Information) from sound to the surface of the device can cause deformation of the film, and the resistance change of the sensor was monitored in real time by an oscilloscope. The original music information can be accurately identified through de‐spectrum analysis. For example, taking the original time domain curve at 50 Hz (Figure S9c,d, Supporting Information) FFT can require a frequency domain curve, which is highly consistent with the original acoustic signal. The outstanding frequency recognition capability of the QCN sensor can be utilized as a high‐accuracy musical instrument tuner. We selected classical music to generate rhythm changes including various frequencies and loudness to investigate the potential of the QCN sensor for rhythm recognition (**Figure** **5**a). The QCN sensor responded in real‐time and captured every feature extraction of music which is beneficial for highly efficient voice recognition (Video S1, Supporting Information). The airflow vibration in the cavity of the orchestral instrument will rapidly excite the real‐time response of the sensor, and the tone of the instrument can be accurately restored from the original signal. As shown in Figure 5b, during repeated three times of the note A4 of the ukulele playing period, the fluctuating frequency of the output signal response remains unchanged and attenuates with the fluctuation of the string. The unique frequency doubling peak of musical (440, 880 Hz) instruments can also be identified in the frequency domain. This phenomenon indicated the potential application of the QCN composites in identifying high‐frequency acoustic waves. Meanwhile, the resistance of the sensor can respond in real‐time, attenuate, and restore stability in a short period of time, and respond immediately during the next test (Figure 5c).

Because of good acoustic distinguishing ability, we recorded detailed sound information while the boy (Henry) and the girl (Cherry) said “Log in” (Figure 5d), the corresponding resistance signal reflects distinctive waveforms and spectrograms. The short‐time Fourier transform (s‐FFT) reflected the acoustic distribution information of the time domain and the frequency domain. From the frequency spectrogram of Figure 5e, the fundamental and harmonic frequencies of the girl are higher and narrower than those of the boy. The different characteristics between these signals can be utilized to distinguish the identity of the voice signal, which may contribute to the smart sensing system and human‐machine interface.

## 3 Conclusion

In summary, we proposed the ultra‐sensitive sensor based on a quasi‐1D conductive network. The FEA simulation revealed the ordered conductive structure can regulate the local stain field and its propagation direction. The strain‐dependent structure change was enlarged along the arrangement direction. The QCN sensor exhibits an ultra‐sensitivity of 862 227 under the strain of 1%. Its performance is far superior to previously reported highly sensitive sensors. Besides, the QCN sensor displays good performance in other key sensing parameters including the minimum detection limit, fast response/recovery time, mechanical stability, and durability. The special QCN structure is sensitive to other mechanical stimulus like compression, bending, shearing, air flow vibration, and weak deformation of the epidermis. Owing to the outstanding sensing performance, the QCN sensor shows potential applications for versatile weak strain detection, human pulse monitoring, spectrum identification, and voice recognition The magnetic field induction fabricating process of the QCN sensor has the features of facile method, good reproducibility, and suitability for mass production. This work demonstrates the feasibility of tuning sensitivity by regulation of conductive networks with heterogeneous structures.

## 4 Experimental Section

### Preparation of QOCN Composites and Ultra‐Sensitive Sensor

First, the thermoplastic polyurethane (TPU) (BASF, 1185A) solution was dissolved by anhydrous dimethylformamide (DMF, 99.5%, Shanghai Aladdin Co., Ltd.) at 15wt% and stirred for 12 h for dispersion. Then, the Ag@Ni MPs (Potters Industries Inc., USA) were mixed with prepared TPU solution by mechanical stirring. Next, the mixture was poured into the customized Teflon molds on the pre‐adjusted horizontal stand. After the surface bubble of the mixture disappeared, the whole magnetization device (Figure S1, Supporting Information) was settled together in the oven curing for 2 h, 60 °C, followed by vacuum drying at 60 °C for 24 h for mechanical reinforcement.

### Characterization and Measurements

The original morphology of the Ag@Ni particles and the cross‐sectional structures of composites were characterized by field‐emission scanning electron microscopy (Sirion 200, FEI). The distribution of elements (Ag and Ni) is identified by the energy‐dispersive X‐ray spectrum (EDS, GENESIS). The top view of the ordered and unordered composites is characterized by optical microscopy (ZEISS Discovery.V12). The electromechanical behavior and sensing property of the sensor were measured at room temperature through a two‐probe configuration, the current source device (Keithley 237, Keithley Instruments, USA) provided electrical input while the voltmeter (Keithley 6517A, Keithley Instruments, USA) illustrated the real voltage value. The oscilloscope (710 110/DLM2024, Yokogawa) was used for high‐frequency acquisition. Stretching and compression tests of the sensors were carried out by a universal material testing machine (Instron 5943, USA). To investigate the response of the ultra‐sensitive sensor to sound vibration, it was settled on the hand‐made box to collect the oscillation surrounding the air. The tones produced by the instrument are calibrated in advance with a commercial tuner to ensure the accuracy of the stimulus signal. The frequency spectrogram of the recording voice signal was processed by taking short‐time FFT through MATLAB software.

### FEM Simulation

The strain field distribution of the quasi‐1D conductive network composites was obtained through a 2D finite element model in the commercial software COMSOL. The size parameter of the geometric model was consistent with the actual device size used for sensing. In the simulation setup, one end was fixed and 1% tensile strain was applied to the other end. The Young's modulus of the soft matrix and conductive particle was considered as 3 MPa and 110 GPa, respectively. The Neo‐Hookean model was used to describe the mechanical response of the TPU matrix, and the conductive Ag@Ni particles adopted the linear elastic model. Considering the densest arrangement of circles in a 2D plane, the average distance between the center circle and the center of the six nearest circles is selected to calculate the overall circle spacing and variation. The same simulation setup for the stretched process was carried out on the compared sample.

## Conflict of Interest

The authors declare no conflict of interest.

## Supporting information

Supporting Information

Supplemental Video 1

## Data Availability

The data that support the findings of this study are available in the supplementary material of this article.
