# Supplementary material for: Quasi‐1D Conductive Network Composites for Ultra‐Sensitive Strain Sensing
Source: Adv Sci (Weinh). 2024 Jun 28;11(33):2403635. doi: 10.1002/advs.202403635 (PMC11434217; doi:10.1002/advs.202403635)
Supplement: Supplementary file 1 — Supporting Information [file ADVS-11-2403635-s001.docx]

**Quasi-one-****dimensional Conductive Network Composites for Ultra-Sensitive Strain Sensing**

*Zhiyi Gao ^a,b^  , Dan Xu ^a,b,c^ , Shengbin Li ^a,b,d^ Ziyin Xiang ^a,b,d^, Haifeng Zhang ^a,b^, Yuanzhao Wu ^a,b^, Yiwei Liu ^a,b,c^, Jie Shang ^a,b,c^* and Run-Wei Li ^a,b,c*^*

**Supporting table:**

**Table S1.**

| **Materials** | **Structure** | **Sensitivity (GF)** | **Lowest** **strain (%)** | **stretching range** | **Reference** |
| --- | --- | --- | --- | --- | --- |
| Ag@Ni/TPU | Quasi-one dimensional | 862227@1% | 0.01 | 0-1% | This work |
| rGO/PDMS | Microcracks | 8699@1% | 0.000064 | 0-1% | ^[31]^ |
| AgNF/PCA | Percolation network | 405.6@5% | **-** | 0-5% | ^[32]^ |
| Graphene/PVDF/TPU | Nanoball decorated porous fiber | 51@5%  87@8% | 0.01 | 0-8% | ^[33]^ |
| Carbon hybrid fiber | Hierarchical fiber | 311@1% | 0.37 | 0-3.1% | ^[34]^ |
| AuNPs-TEG/PI | 3D covalent network | 126@0.8% | - | 0-0.6% | ^[35]^ |
| rGO/PDMS | gradient wrinkle | 19.24@8% | 0.1 | 0-300% | ^[36]^ |
| ITO/PET | Crack | 4000@2% | - | 0-2% | ^[37]^ |
| Au/PDMS | Microcrack arrays | 5888.89@2% | - | 0-2% | ^[38]^ |
| ITO/PU | Micromesh | 744.3@2% |  | 0-2% | ^[39]^ |

**Supporting Note 1**. The definition and calculation of the distance change factor (***DF***).

We considered that the tightest packing arrangement of circles in a two-dimensional plane is shown in the figure below:


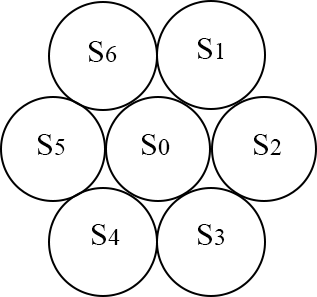


Thus, we take the average distance between the center circle ($S_{0}$) and its nearest six circles ($S_{1}\sim S_{6}$) as the average distance ($D_{ave}^{j}$) among the circle and the surrounding circle. As shown in the following figure, the distances between the six centers closest to the center circle are $d_{\min}^{1}\sim d_{\min}^{6}$, thus $D_{ave}^{j}=\frac{\sum_{1}^{6} d_{\min}^{i}}{6}$.($d_{min}^{7}$ is larger than $d_{\min}^{1}\sim d_{\min}^{6}$, which is not included in$D_{ave}^{j}$)

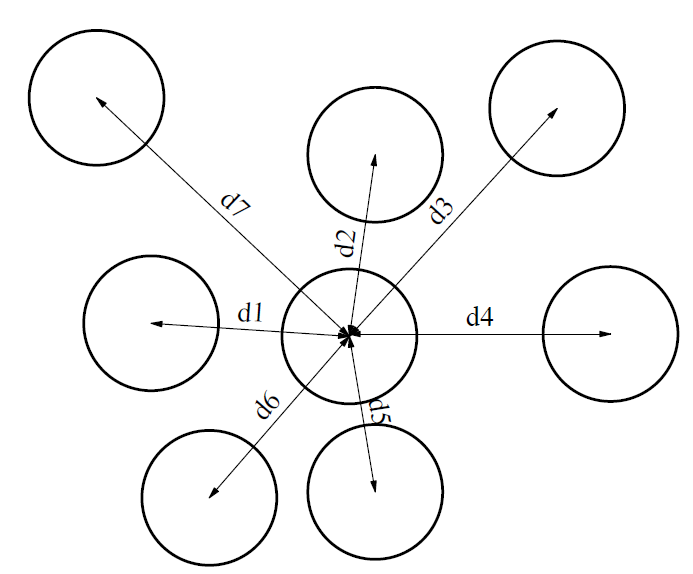


By calculating and averaging $D_{ave}^{j}$of all circles in the plane, the whole circle spacing $D_{all}$ is obtained，the specific formula is as follows:

$D_{\text{ave}}^{j}=\frac{\sum_{1}^{6} d_{\min}^{i}}{6}$ (1)

$D_{all}=\frac{\sum_{1}^{n} D_{ave}^{j}}{n}$ (2)

The distance change factor (***DF***) is defined as:

$\frac{D_{all}}{D_{all,init}}$ (3)

$D_{all}$ represents the overall circle spacing for each stretching stage，$D_{all,init}$ represents the initial circle spacing when the strain is 0 %.

**Supporting figures:**

**
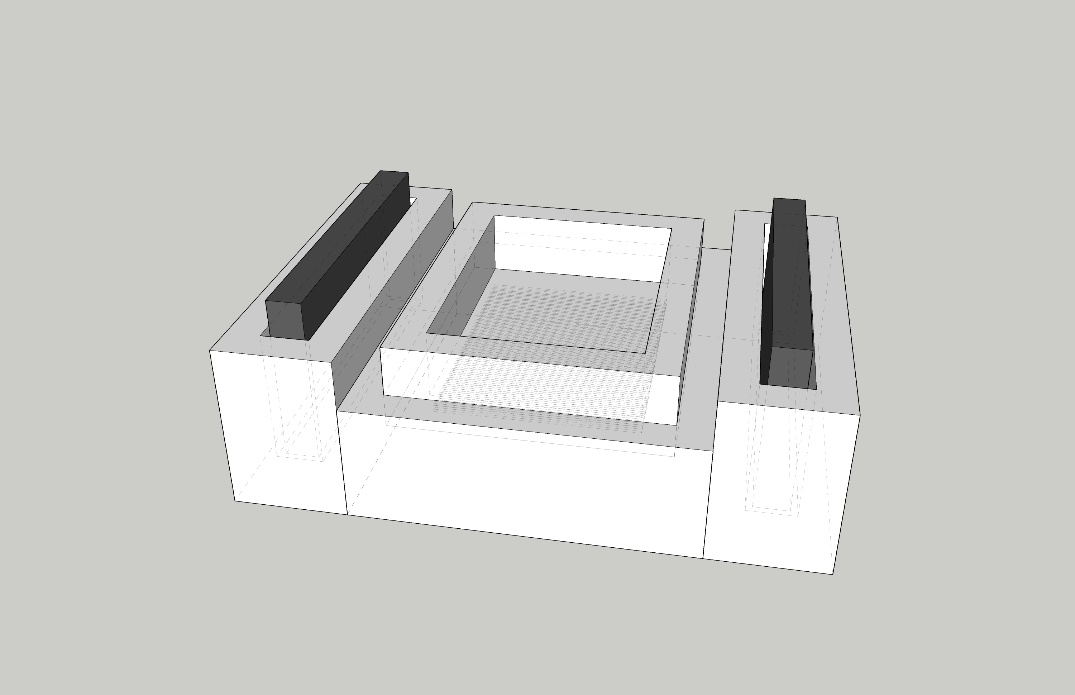
**

Figure S1. Schematic diagram of the structure of the magnetization device. The left and right ends are the magnet fixing devices that can be removed in real time, and the mold can be fixed at the center of the parallel plate magnet.


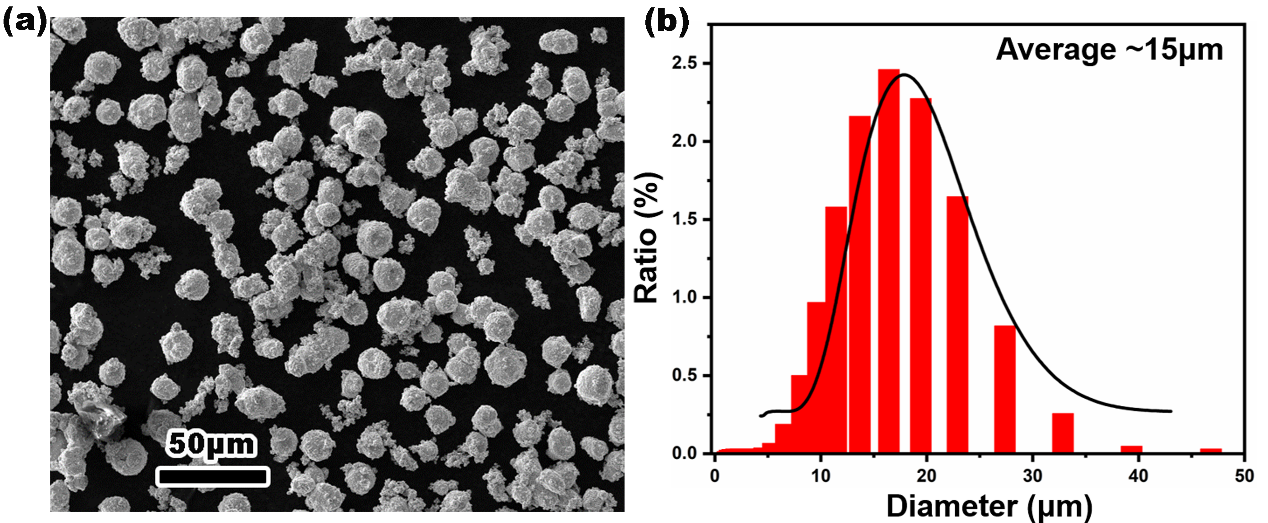


Figure S2. Characterization of the Ag@Ni MPs. (a) SEM image of the Ag@Ni MPs. The whole particles are well coated by silver, which keep the spherical shape at the same time. The resistivity of the MPs is around 0.55 mΩ.cm. (Scale bar: 50μm) (b) The average diameter of the MPs is around 15μm.


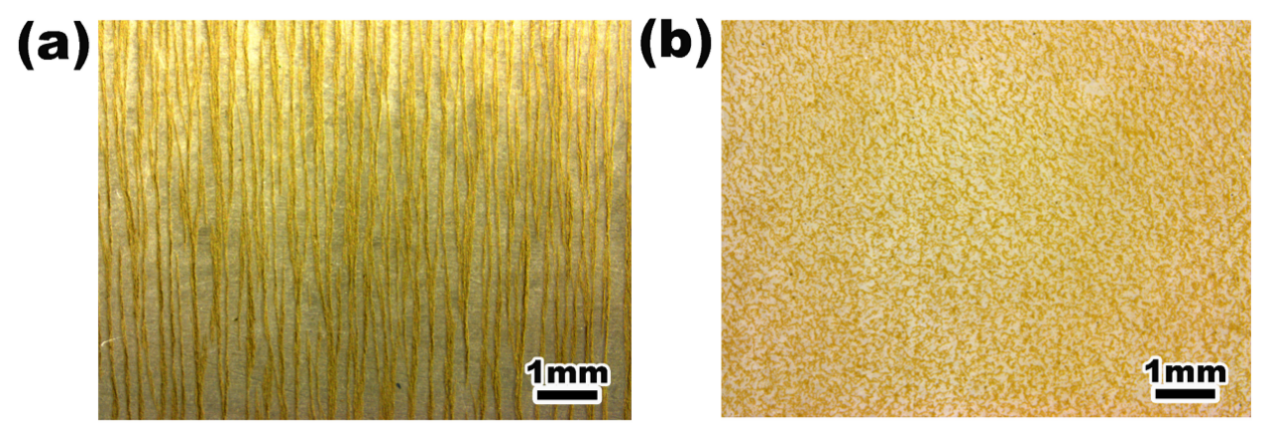


Figure S3. Optical microscope images of the top side view of QCN composites. (a) Ordered (b) Unordered. The direction of the chain orientation is the direction in which the magnetic field is applied. There existed a clear separation distance between the conductive paths formed by the conductive fillers in the QCN composites.


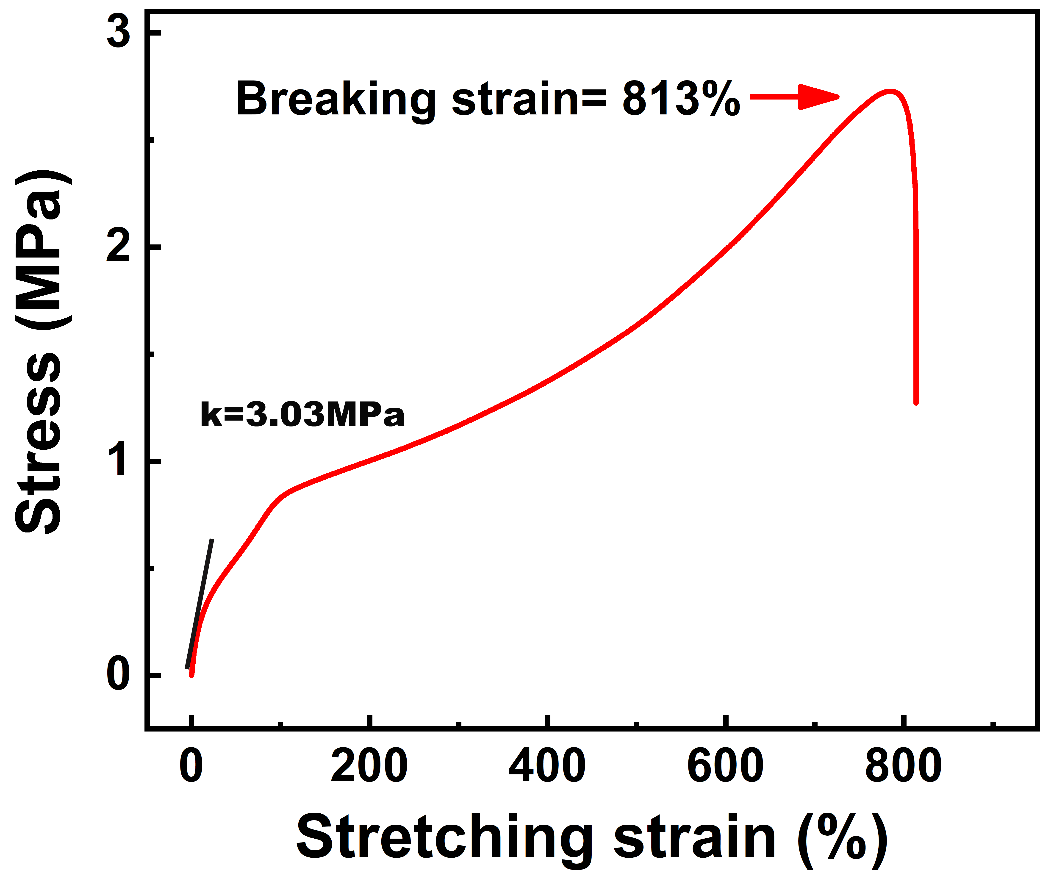


Figure S4. Stress-strain curve of the QCN composites evaluated under strain rate of 60mm/min. Young’ s modulus of the QCN composites is estimated to be about 3.03 MPa, calculated from the slope of the stress-strain curve. The break elongation strain of the QCN composites is about 813%.


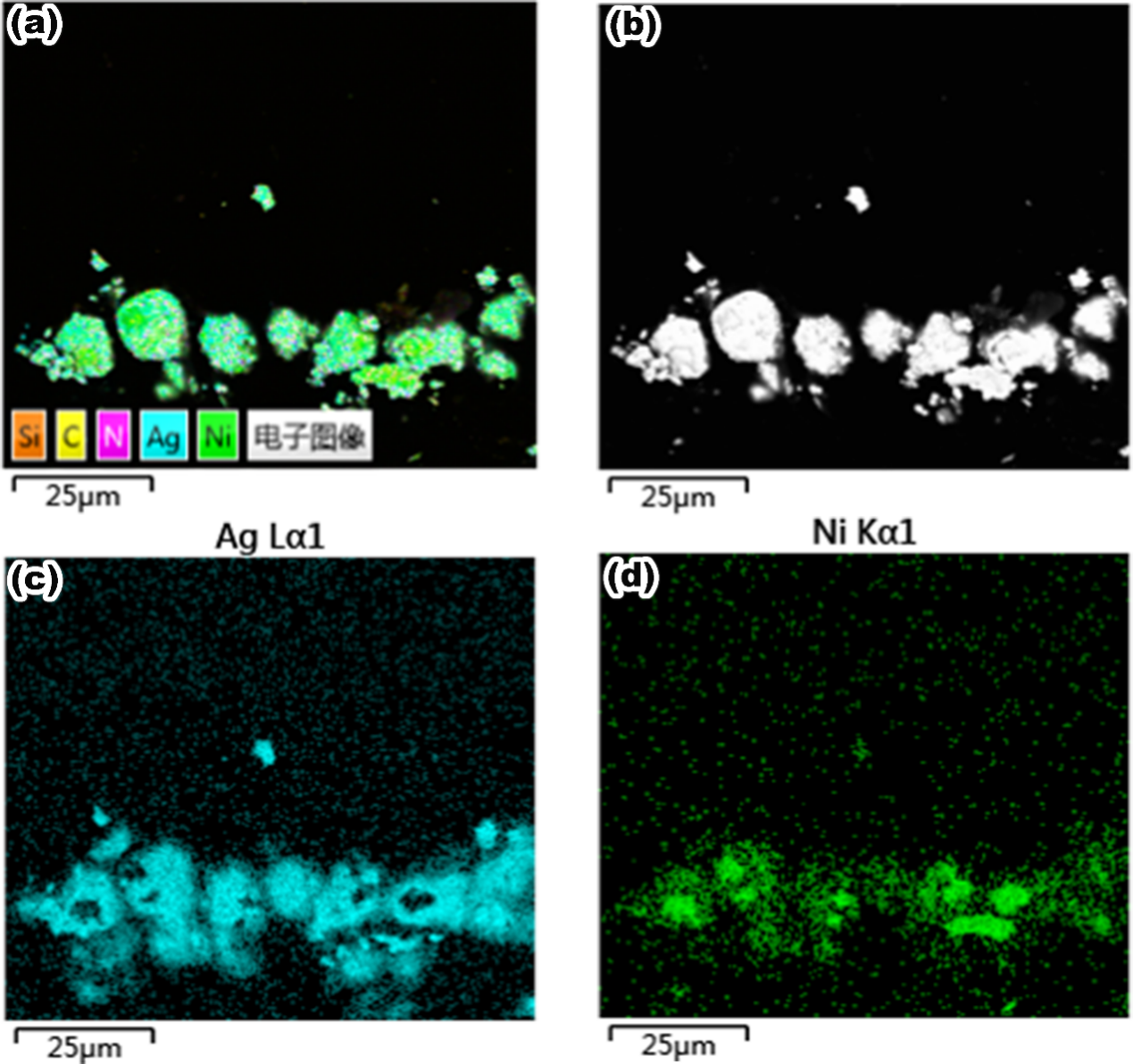


Figure S5. Characterization of the QCN composites. (a-d) The SEM images and EDS mappings of the QCN composites at the cross view. (Ag. Ni). Every single element oriented obviously as a 1D chain along each magnetic field.


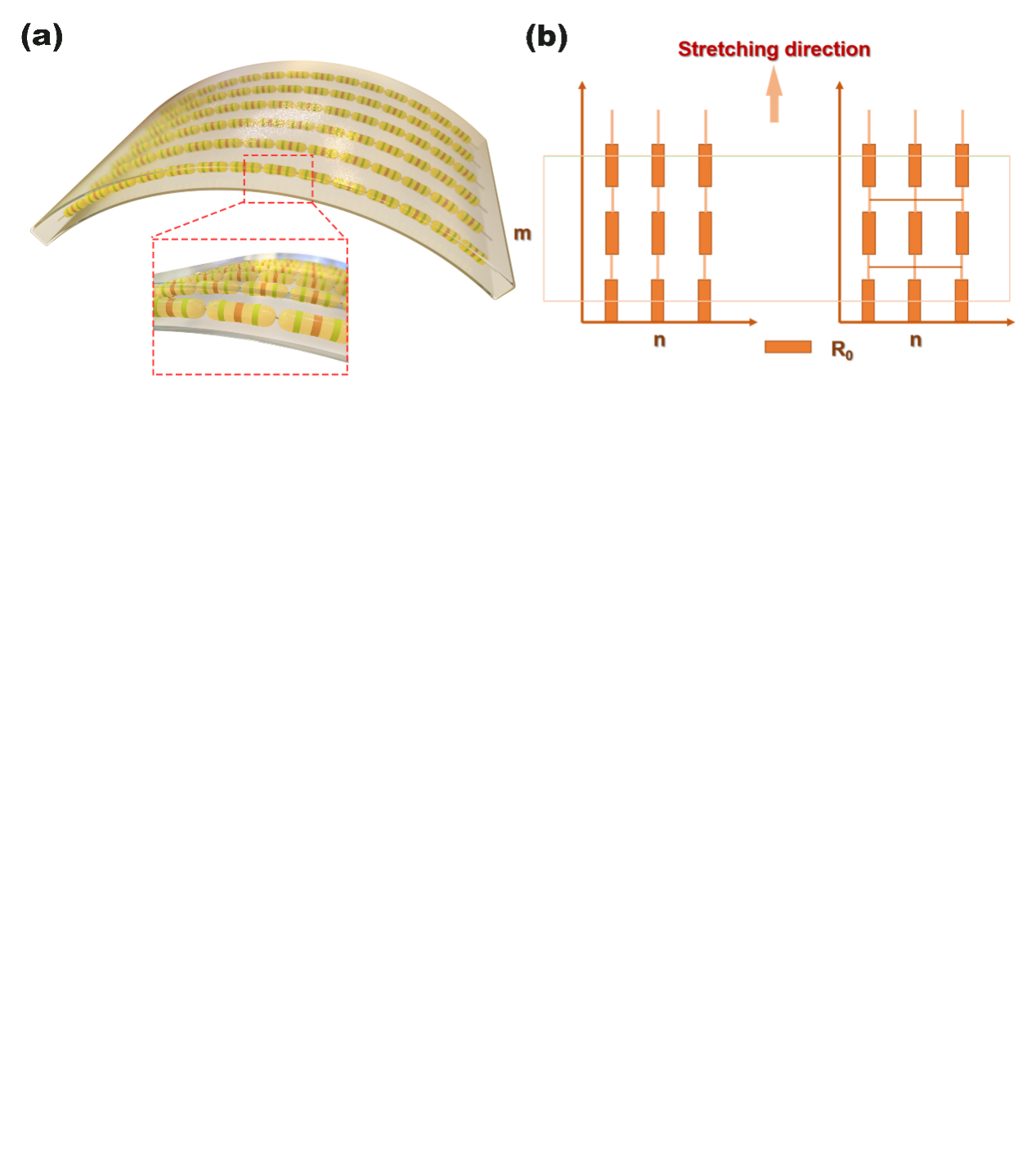


Figure S6. (a) The Schematic structure diagram of the QCN sensor. (b)Schematic of the Equivalent Circuit about the ordered and unordered conductive network under stretching.

The electrical resistance variations of the AGCF were dominated by the increase in crack number and area. Here, a simplified model was proposed to further illustrate the crack based piezoresistive sensing mechanism (Figure S11).20,47 The network consisting of m × n unit cells represented the total electrical resistance of the generated hierarchical cracks. The equivalent electrical resistance of each cell crack (i, j) was denoted as Ri,j, and the total resistance of all cracks was taken as a function of Ri,j, where the crack number was denoted as N, and the average area of the cracks was denoted as S. In general, the resistances of the cracks were much larger than those of the connected regions on the film. Thus, the total electrical resistance of the film could be regarded as proportional to N × S. Since the total crack area increased linearly with strain loading, the relative change of total electrical resistance can be demonstrated to be proportional to the external strain, which exactly revealed the linear sensing mechanism of the AGCF strain sensor.





Figure S7. The relative resistance changes of the QCN composites based ultra-sensitive sensor during repeated stretching for 1000 times, at the strain of 0.2%. At the beginning 10 times of the fatigue test, the average $\Delta R/R_{0}$ was higher than the last 10 times, which may cause by the limitation of the electrical detection instruments and polymer substrate induced fluctuation.


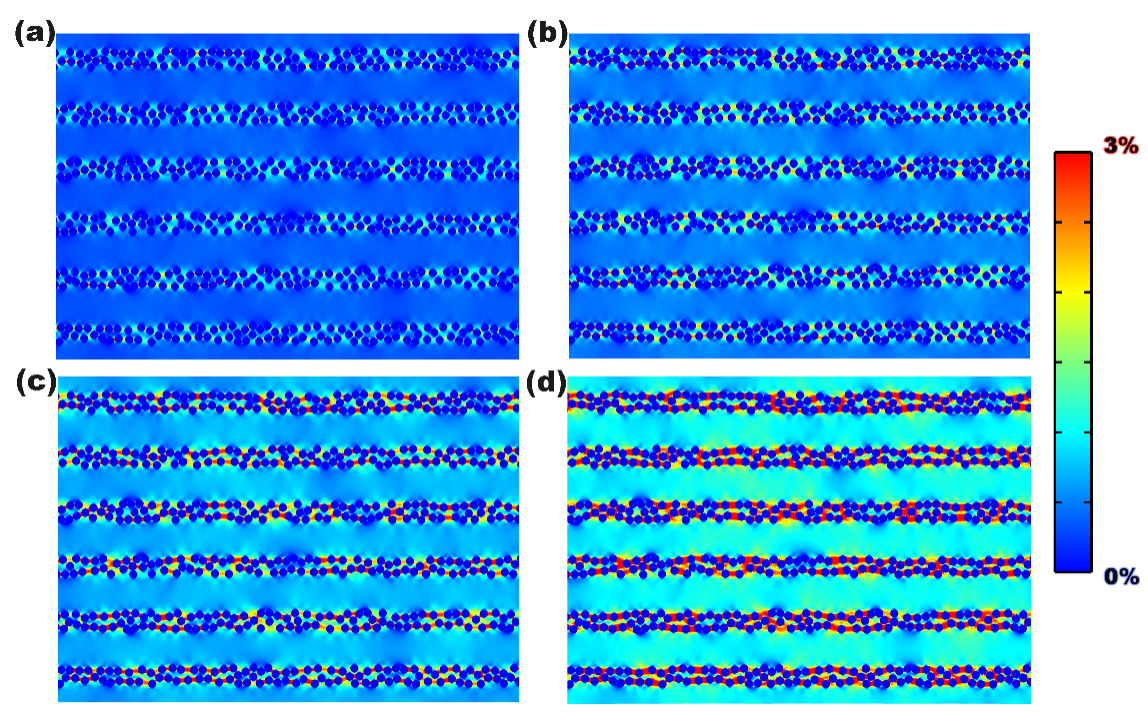


Figure S8. The dynamic strain distribution during the loading process under 1%. Each cloud map was selected at equal time intervals, and the highest real strain was up to 3%.


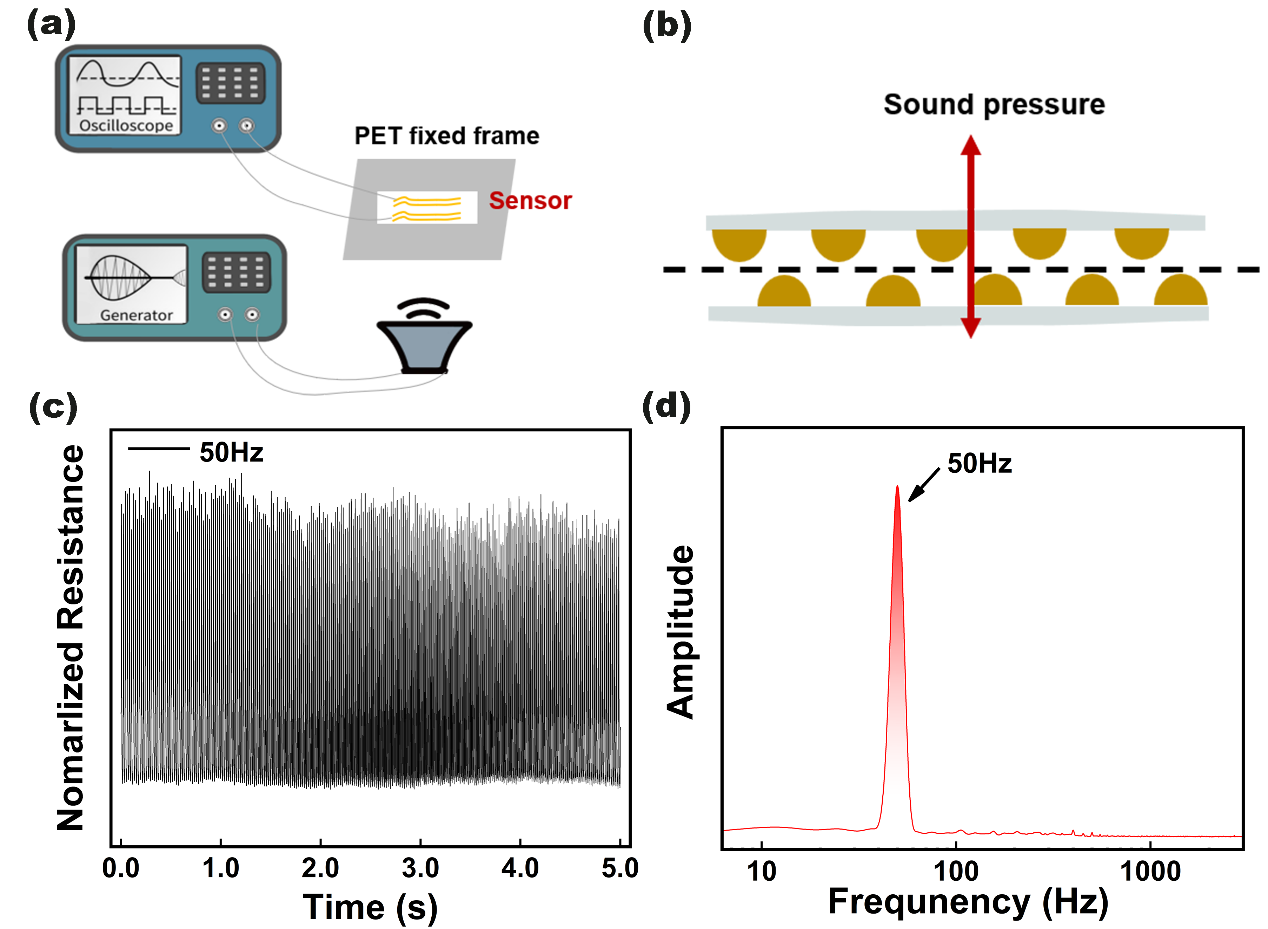


Figure S9. Accurate recognition ability of the sinusoidal audio. (a) Schematic diagram of acoustics detection device. (b) The force diagram of the QCN based sensor under sound pressure. (c) Original resistance response at the frequency of 50Hz. (d) The frequency domain spectrum of the output signal by taking FFT transform.





Figure S11. Real-time resistance changes during continuous three wave string fluctuations. The resistance response kept changing in good sync with note presentation.

**References**

[1]. Dinh Le, T. S. et al. Ultrasensitive anti-interference voice recognition by bio-inspired skin-attachable self-cleaning acoustic sensors. *ACS Nano* **13**, 13293-13303 (2019).

[2]. Yang, Y. et al. A naturally-derived supramolecular elastomer containing green-synthesized silver nanofibers for self-repairing e-skin sensor. *Journal of Materials Chemistry C* **7**, 578-585 (2019).

[3]. Huang, T. et al. Porous fibers composed of polymer nanoball decorated graphene for wearable and highly sensitive strain sensors. *Advanced Functional Materials* **29**, (2019).

[4]. Hu, Y. et al. Ultrasensitive and wearable carbon hybrid fiber devices as robust intelligent sensors. *ACS Appl Mater Interfaces* **13**, 23905-23914 (2021).

[5]Huang, C. B. et al. Highly sensitive strain sensors based on molecules-gold nanoparticles networks for high-resolution human pulse analysis. *Small* **17**, e2007593 (2021).

[6]. Chu, Z. et al. Superhydrophobic gradient wrinkle strain sensor with ultra-high sensitivity and broad strain range for motion monitoring. *Journal of Materials Chemistry A* **9**, 9634-9643 (2021).

[7] Lee, T. et al. Transparent ito mechanical crack-based pressure and strain sensor. *Journal of Materials Chemistry C* **4**, 9947-9953 (2016).

[8] Han, Z. et al. High-performance flexible strain sensor with bio-inspired crack arrays. *Nanoscale* **10**, 15178-15186 (2018).

[9] Qiao, Y. et al. Intelligent and highly sensitive strain sensor based on indium tin oxide micromesh with a high crack density. *Nanoscale* **14**, 4234-4243 (2022).
